# Epigenetic profiling reveals M1 macrophages as the primary drivers of trained immunity in HSV-1 latently infected mice

**DOI:** 10.1371/journal.ppat.1014598

**Published:** 2026-09-09

**Authors:** Ujjaldeep Jaggi, Shaohui Wang, Homayon Ghiasi

**Affiliations:** >Center for Neurobiology & Vaccine Development, Cedars-Sinai Health Sciences University, Ophthalmology Research, Department of Surgery, Los Angeles, California, United States of America; Oklahoma State University College of Osteopathic Medicine, UNITED STATES OF AMERICA

## Abstract

Macrophages play multifaceted and critical roles in controlling diverse pathogenic infections. Building on our recently published observations of macrophages exhibiting memory responses to HSV-1 infection, our current report investigates the macrophage subtype responsible for generating trained immunity against virus-induced immunopathogenesis. Using ATAC-seq (Assay for Transposase-Accessible Chromatin using sequencing), an epigenetic profiling technique, we identified chromatin accessibility changes in both M1 and M2 macrophages associated with the acquisition of IRGM1, a marker of the trained phenotype. To conduct this study, we first generated M0, M1, and M2 macrophage subtypes from bone marrow (BM) derived macrophages isolated from HSV-1 latently infected wild type (WT) mice. ATAC-seq revealed that M1-generated macrophages displayed higher IRGM1-associated chromatin accessibility peaks compared to M2 and M0 subtypes, and this response was enhanced after stimulation with UV-inactivated virus. To further dissect this response, we analyzed memory responses in bone marrow-derived macrophages, spleen macrophages, corneal macrophages, and trigeminal ganglia (TG) of latently infected M1 and M2 macrophages. Flow cytometry and ATAC-seq data showed a significantly higher proportion of IRGM1 ⁺ macrophages in infected M2^-/-^ mice, which are enriched in M1 macrophages. These findings indicate that M1 macrophages, but not M2 macrophages, undergo trained immunity in response to HSV-1 infection. This is also confirmed by the Luminex assay, in which M1 macrophages, after stimulation, then known as primed M1 macrophages, enhance the secretion of pro-inflammatory cytokine/chemokine response to secondary HSV-1 exposure. These results uncover a previously underappreciated role for macrophage-mediated trained immunity in antiviral defense against HSV-1 infection and offer new insights into potential therapeutic targets for modulating host immune responses during HSV-1 infection.

## Author summary

HSV-1 is a widespread pathogen that establishes lifelong infection and can cause recurrent disease and severe immunopathology. It is well established that T cells develop memory responses to target pathogens, but little is known about whether macrophages can also develop trained immunity, which remains poorly understood in the context of viral infections. Our current report identifies M1 macrophages as the key subset responsible for acquiring trained immunity during HSV-1 infection, characterized by epigenetic reprogramming and increased accessibility of IRGM1-associated chromatin regions. In contrast, M2 macrophages do not exhibit this trained phenotype. These findings redefine the functional specialization of macrophage subsets in a previously unrecognized mechanism by which innate immune cells contribute to long-term host protection. By highlighting M1 macrophages as drivers of trained immunity, this work opens new avenues for therapeutic strategies to enhance antiviral responses while limiting immunopathology.

## Introduction

Viruses have evolved over several thousand years to be highly successful predators of living cells and have learned to manipulate host defense mechanisms. Thus, viruses employ various stealth invasion strategies, such as antigenic drift, resistance to immune effector mechanisms, and immune response suppression [1]. One such evasion approach is adopted by herpesviruses, which enter latency to evade immune detection. Herpes simplex virus type 1 (HSV-1) is a highly intriguing and resilient virus that can periodically reactivate from latency, causing Herpes Stromal Keratitis (HSK), which is an immunopathological disease [2–4]. Previously, we reported that macrophages are among the dominant cellular infiltrates in the eyes of ocularly infected mice and appear as early as 1 hr post infection (PI) [5]. Furthermore, in our recently published report, we showed that, similar to T cells, natural killer (NK) cells, dendritic cells (DCs), group 2 innate lymphoid cells (ILC2s), and B cells, macrophages also have memory towards HSV-1 infection as determined by upregulation of IRGM1 (immunity-related GTPase family M protein) response using ATAC-Seq [6]. IRGM1 regulates the motility of M1 macrophages [7]. Furthermore, IRGM1 expression in M1 macrophages, but not in M2 macrophages, is known to regulate chromatin remodeling and histone modifications during M1 macrophage activation [8]. Overall, control of homeostasis and cytokine production in macrophages is thought to be the primary mechanism by which IRGM/IRGM1 controls inflammatory diseases [9].

One of the most remarkable characteristics of macrophages is their plasticity and heterogeneity [10–12]. Our published studies demonstrated that: 1) macrophage depletion increases virus replication in the eyes and trigeminal ganglia (TG) of infected mice [13]; 2) injection of WT mice with M-CSF or IL-4, or their infection with a recombinant HSV-1 expressing IL-4 (HSV-IL-4), pushes macrophages toward an M2 response, whereas injection of mice with IFN-γ or their infection with an IFN-γ expressing recombinant virus (HSV-IFN-γ) enhances an M1 response [14]; 3) recombinant HSV-1 expressing IL-4 (an inducer of M2 responses) provided better protection than a recombinant HSV-1 expressing IFN-γ (an inducer of M1 responses) or wild type (WT) virus against virus replication, latency, reactivation, and eye disease in WT type mice [15]; 4) using M2-knockout mice (M2^-/-^ mice) and M2 transgenic mice (overexpressing M2 macrophages) showed that increased M2 macrophages correlated with increased phagocytosis, increased primary virus replication, and increased latency. In contrast, the absence of M2 macrophages did not significantly alter HSV-1 infectivity from that of WT mice [16]; 5) lack of M1 macrophages in M1^-/-^ mice affects HSV-1 infectivity and demonstrated the importance of M1 macrophages specifically in controlling primary virus replication, eye disease, and survival, but not latency-reactivation [17]; and 6) blocking autophagy in M1 macrophages increased both virus replication in the eyes and eye disease in comparison to blocking autophagy in M2 macrophages or WT control mice, but blocked autophagy did not affect latency-reactivation [18].

Recently, we have shown that macrophages have memory responses to HSV-1 infection [6]. Thus, in this study, we investigated which subset of macrophages is responsible for this memory response. Therefore, we examined the roles of M1 and M2 macrophages in immune training. Our data demonstrated that M1-derived macrophages from WT mice showed a significantly higher peak in IRGM1 expression than M2-derived macrophages. Similarly, M2^-/-^ mice (with M1 macrophages) showed higher IRGM1 peak expression by ATAC-seq than M1^-/-^ mice (with M2 macrophages). Furthermore, we confirmed our results using flow cytometry, which showed that M1 macrophages exhibit a significant memory response following HSV-1 infection in their spleen, cornea, and TG. Moreover, our Luminex results suggest a strong correlation between macrophage training and cytokine/chemokine response. Collectively, these findings suggest that latent infection primes M1 macrophages to acquire a trained, memory-like phenotype, resulting in enhanced production of pro-inflammatory cytokines and chemokines following secondary stimulation and potentially contributing to improved innate host defense. These findings provide the first evidence that M1 macrophages can exhibit a memory-like response to HSV-1 infection.

## Materials and methods

### Ethics statements

All procedures were performed in strict accordance with the Association for Research in Vision and Ophthalmology Statement for the Use of Animals in Ophthalmic and Vision Research and the NIH Guide for the Care and Use of Laboratory Animals (ISBN 0-309-05377-3). The animal research protocol was approved by the Institutional Animal Care and Use Committee of Cedars-Sinai Medical Center (protocol no. 8837).

### Mice and viruses

Six-to-seven-week-old male and female WT C57BL/6J mice, as well as conditional STAT1^-/-^ and GATA3^-/-^ mice, were used in the experiments. STAT1^-/-^ and GATA3^-/-^ mice that are lacking expression of M1 and M2 in their macrophages, respectively, are designated as M1^-/-^ and M2^-/-^ as described previously and being used in this study [16,17]. WT C57BL/6J control mice were purchased from The Jackson Laboratory (Bar Harbor, ME) and bred in-house at Cedars-Sinai Medical Center. WT C57BL/6J mice were infected ocularly with 2 x 10^5^ pfu/eye of Plaque-purified, virulent HSV-1 strain McKrae, without corneal scarification, and M1^-/-^ and M2^-/-^ mice were infected with avirulent HSV-1 strain KOS, with corneal scarification. Control mice for each strain of mice were mock-infected.

### Preparation and stimulation of macrophages from bone marrow (BM)

Femoral bones were dissected, each bone end was cut off, the BM was isolated, cells were cultured for 6 days and further differentiated and activated as described [16,17]. WT BM-derived macrophages were seeded at 2 X 10^5^ cells per well in a 24-well plate, and on day 6 post-generation, macrophages were scraped out, counted, and plated for M1 and M2 differentiation as described previously [16,17]. The following day, cells were treated with 10 pfu/cell of the UV-inactivated HSV-1 strain McKrae and incubated for 1 hr. Treated or mock-treated cells were then washed three times with PBS, and fresh complete DMEM was added to each well for 24 hr. Cell monolayers were harvested and subjected to ATAC-seq and Flow cytometry.

### ATAC-sequencing (ATAC-seq)

Macrophages were generated from BM or isolated from the spleens of infected mice, as described below. BM-derived macrophages and isolated spleen macrophages were stimulated with UV-inactivated virus, and 24 hr post-stimulation, cells were harvested and subjected to ATAC-seq performed by the Applied Genomics, Computation, and Translational Core at Cedars-Sinai Medical Center, Los Angeles, California, USA, as described [6].

### Luminex xMAP immunoassay (magnetic bead kits)

Luminex assays were performed in the Immune Assessment Core at the University of California, Los Angeles (UCLA, CA). Mouse 32-plex magnetic cytokine/chemokine kits were purchased from EMD Millipore (Billerica, MA) and used according to the manufacturer’s instructions. Unpolarized (MO), M1 or M2 macrophages were generated from BM and on day 7 post-generation, cells were stimulated and non-stimulated with 10 pfu/cell of UV-inactivated McKrae virus for 24 hr. Media from the infected and mock-infected cells were collected, and 25 μl of 1:2-diluted samples were mixed with 25 μl of magnetic beads and incubated overnight at 4°C with shaking. After washing the plates twice with wash buffer in a Biotek ELx405 washer, 25μl of biotinylated detection antibody was added and incubated for 1 hr at room temperature. Streptavidin-phycoerythrin conjugate (25 μl) was then added to the reaction mixture and incubated for another 30 mins at room temperature. Following two washes, beads were resuspended in sheath fluid, and fluorescence was quantified using Luminex.

### Flow Cytometry and cell sorting analysis

Were performed on BM–derived macrophages, spleen macrophages, TG, and corneal cells from infected and mock-infected mice. Data acquisition was conducted using a BD Symphony A5 Cell Analyzer, and analysis was performed with FlowJo software. Cell sorting was carried out using a BD Aria III sorter. Spleens were harvested on day 35 PI, minced, and digested in 1 × HBSS containing collagenase D (2 mg/mL) and DNase I (20 U/mL) at 37°C for 30 minutes. Single-cell suspensions were prepared by mechanical dissociation through a cell strainer, followed by red blood cell lysis using 1 × RBC lysis buffer (Sigma) for 3 minutes at room temperature. Cells were washed with cold PBS, blocked with TruStain FcX (BioLegend), and stained with antibodies APC-CD11b (BioLegend, 101212), PE-F4/80 (BioLegend, 123110), FITC-CD3 (BioLegend, 100204), FITC-Ly6G (BioLegend, 127606), and FITC-CD19 (BioLegend, 115506). Macrophages were sorted based on CD11b⁺F4/80 ⁺ expression. For intracellular IRGM1 detection, cells were stained for surface markers (CD45 and F4/80), then fixed, permeabilized, and stained with Alexa Fluor 647 conjugated anti-IRGM1 antibody according to the manufacturer’s protocol. In parallel, total spleen-derived single-cell suspensions were stained for CD45 ⁺ F4/80 ⁺ macrophages and were sorted and used for RT-PCR analysis.

BM cells were isolated from latently infected and mock-infected mice and cultured with macrophage growth factors as described above. On day 6 PI, differentiated macrophages were harvested, replated, and stimulated with UV-inactivated HSV-1 strain McKrae (10 pfu/cell) for 1 hr, followed by collection at 24 hr post-stimulation. Cells were stained with antibodies against BV421-CD45 and PE-F4/80, along with lineage markers (FITC-CD3, FITC-CD19, and FITC-Ly6G) and the viability dye 7-AAD. Flow cytometric analysis was performed, and macrophages were also collected for RT-PCR analysis.

Corneas from latently infected and mock-infected mice were pooled, minced, and digested in RPMI containing 10% FBS, 1.25 mg/mL Liberase (Roche), and RNase-free DNase (Qiagen) at 37°C for 1 hour to generate single-cell suspensions. Cells were blocked with mouse TruStain FcX (BioLegend) at 1 μg/10^6^ cell to prevent nonspecific Fc receptor binding, then stained with BV421-CD45 and PE-F4/80 antibodies (BioLegend) according to the manufacturer’s protocol. For intracellular IRGM1 detection, cells were fixed, permeabilized, and stained with AF647-conjugated anti-IRGM1 antibody. Samples were analyzed by flow cytometry, and CD45 ⁺ F4/80 ⁺ IRGM1 ⁺ macrophages were quantified and compared between infected and mock-infected groups. Similarly, TG from latently infected and mock-infected mice were harvested, dissociated into single-cell suspensions, and subjected to flow cytometry. Cells were stained for F4/80^+^IRGM1^+^ macrophages, and IRGM1 expression was evaluated for both M1 and M2 macrophages.

### Statistical analysis

For all statistical tests, p-values less than or equal to 0.05 were considered statistically significant and marked by a single asterisk (*). P-values less than or equal to 0.001 were marked by double asterisks (**). A two-tailed Student’s t-test with unequal variances was used to compare differences between two experimental groups. A one-way ANOVA test was used to compare differences among three or more experimental groups. Graphs were plotted using GraphPad Prism 9 software. All experiments were repeated at least three times to ensure accuracy.

## Results

### M1 macrophages have higher IRGM1 chromatin accessibility than M2 macrophages from latently infected WT mice using ATAC analysis

**Fig 1:**
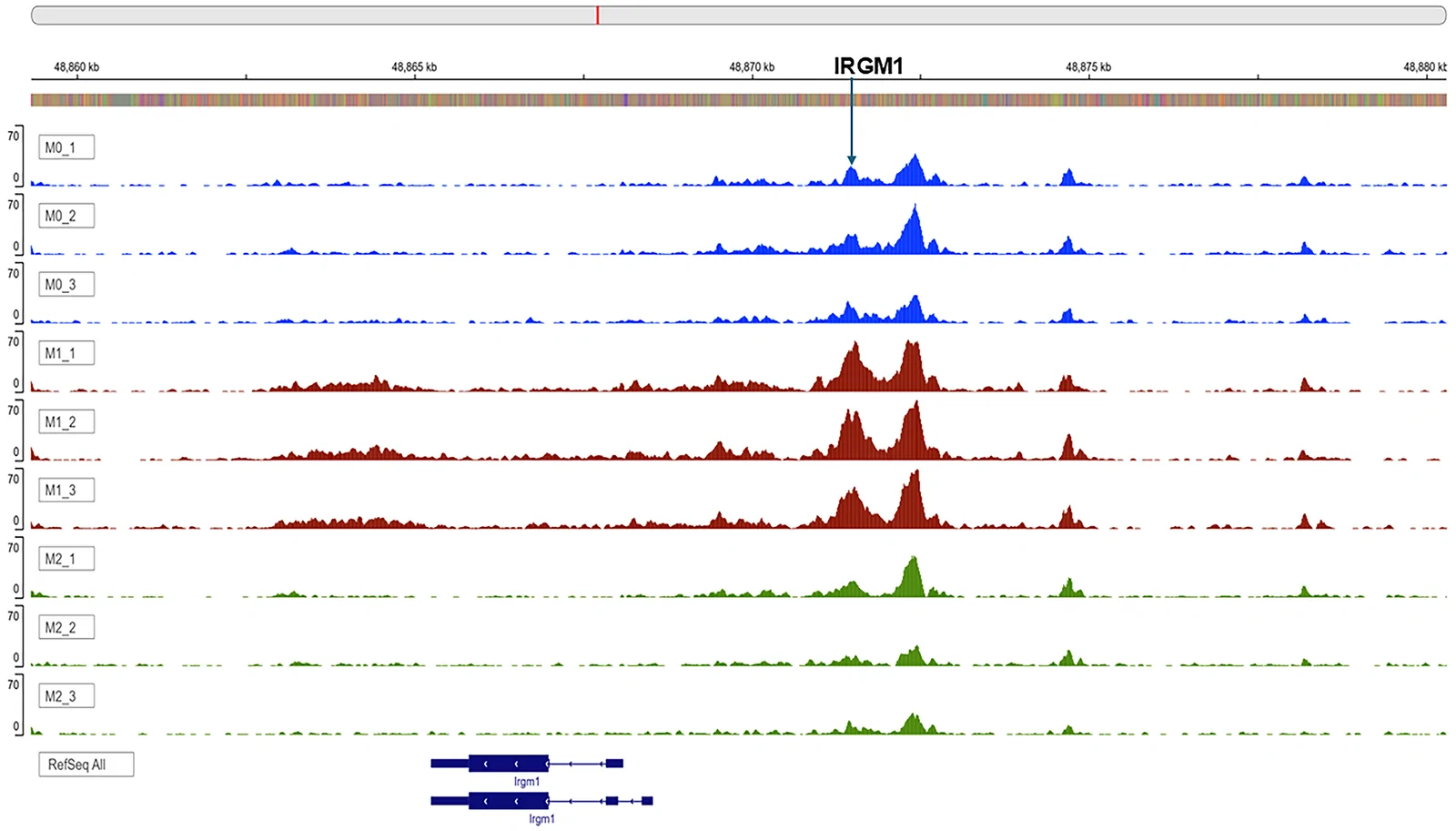
IRGM1 expression in WT mice BM-derived macrophages. WT mice were infected ocularly with 2 X 10^5^ PFU/eye of HSV-1 strain McKrae without corneal scarification. On day 35 PI, BM from infected mice was harvested and polarized *in vitro* using IFN-γ to generate M1 macrophages, IL-4 to generate M2 macrophages, or M0 as a control. M0, M1, and M2 macrophages were stimulated for 24 hr with 10 pfu/cell of UV-inactivated HSV-1 strain McKrae. BM-derived M0, M1 and M2 stimulated macrophages, were stained with antibodies against CD3, CD19, Ly-6G, F4/80, CD45, or 7-AAD. The CD45^+^F4/80^+^CD3^-^CD19^-^Ly6g^-^ macrophages were sorted and subjected to ATAC-seq. Blue-colored IRGM1 peaks represent the M0 macrophage population, red indicates M1-generated macrophages, and green indicates M2-generated macrophages. Peaks of open chromatin were visualized by the IGV web browser (UCSD).

WT mice were infected ocularly with 2 X 10^5^ pfu/eye of HSV-1 strain McKrae without corneal scarification. On day 35 PI, BM from infected mice was harvested, and macrophages were generated as previously described [16,17]. On day 6 post-isolation, macrophages were polarized *in vitro* using IFN-γ to generate M1 macrophages or IL-4 to generate M2 macrophages, as described [14,15], with M0 as the control group. After 24 hr post-polarization, M0, M1, and M2 macrophages were harvested and stimulated with 10 pfu/cell of UV-inactivated HSV-1 strain McKrae. After 24 hr post UV-stimulation, BM-derived macrophages were subjected to ATAC-seq. IRGM1 peak levels were elevated in stimulated BM-derived macrophages compared to unstimulated BM-derived macrophages **(**Fig 1**, arrow indicates IRGM1 peaks).**

M1-generated macrophages had a higher peak of IRGM1 chromatin accessibility than did M2-generated macrophages (Fig 1). A lower peak was detected in M2-infected macrophages than in M1-infected macrophages (Fig 1). We observed a peak in M2 infected-stimulated macrophages. However, it was still smaller than in the M1 infected-stimulated group (Fig 1). The peak in M2 infected-stimulated macrophages is probably due to the presence of some M1 macrophages in this group. We also observed the IRGM1 peak in the M0-infected group, but, as before, the possibility of M1 macrophages present during macrophage generation remains. Overall, the peak of IRGM1 was significantly higher in M1 macrophages than M2 or M0 infected macrophages (Fig 1).

### Lower IRGM1 chromatin accessibility is associated with the absence of M1 macrophages in M1^-/-^ latently infected mice

**Fig 2:**
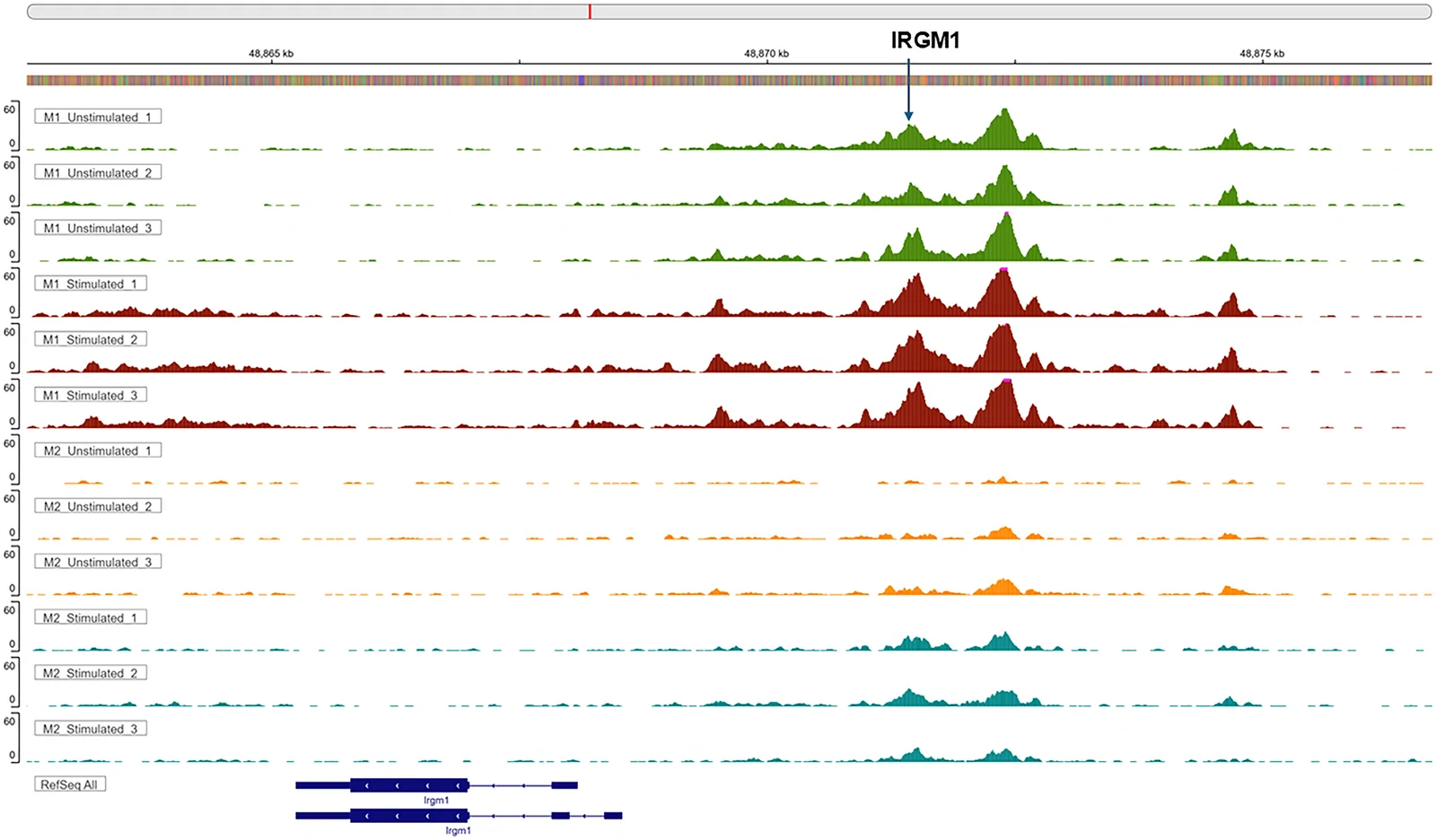
Role of M1 macrophages in memory response to HSV-1 infection using M1^-/-^ and M2^-/-^ mice. M1^-/-^ and M2^-/-^ mice were ocularly infected with 2 X 10^5^ PFU/eye of HSV-1 strain KOS with corneal scarification. On day 35 PI, BM was isolated, and macrophages were generated and stimulated with UV-inactivated virus. After 24 hr PI, macrophages underwent ATAC-seq analysis. IRGM1 peaks in green represent the M1 Unstimulated group; red IRGM1 peaks signify the M1 stimulated group; orange peaks represent the M2 unstimulated macrophage population; and teal blue denotes the M2 stimulated group. Peaks of open chromatin were visualized by the IGV web browser (UCSD).

The above results with M0, M1 and M2 BM-derived macrophages from WT mice showed that IRGM1 chromatin accessibility peaked in M1 macrophages in comparison to M2 macrophages (Fig 1). Next, to confirm the importance of M1 macrophages to HSV-1 memory, we infected M1^-/-^ mice (lacking M1 macrophages but having M2 macrophages) and M2^-/-^ mice (lacking M2 macrophages but having M1 macrophages) mice with 2 X 10^5^ pfu/eye of HSV-1 strain KOS, with corneal scarification, as described in Materials and Methods. In this experiment, we used the avirulent HSV-1 strain KOS rather than the virulent strain McKrae because M1^-/-^ mice are highly susceptible to the virulent strain McKrae [17]. On day 35 PI, BM was isolated, and macrophages were generated from M1^-/-^ and M2^-/-^ mice, which were stimulated with UV-inactivated virus as described above. The IRGM1 chromatin accessibility peak with and without stimulation was higher in M2^-/-^ mice, which have M1 macrophages, than in M1^-/-^ mice that have M2 macrophages (Fig 2**, arrow indicates IRGM1 peaks)**. Macrophages from latently infected M2^-/-^ mice responded *in vitro* to antigen stimulation with increased IRGM1 peak **(**Fig 2**, M2**^**-/-**^ **infected-stimulated, compared with M1**^**-/-**^ **infected-stimulated).** These results suggest that the IRGM1 regulatory network in M1 macrophages is epigenetically reprogrammed towards memory response during infection and can change substantially upon re-stimulation.

### ATAC-seq shows higher IRGM1 expression in the spleens of infected M2^-/-^ mice with intact M1 macrophages

**Fig 3:**
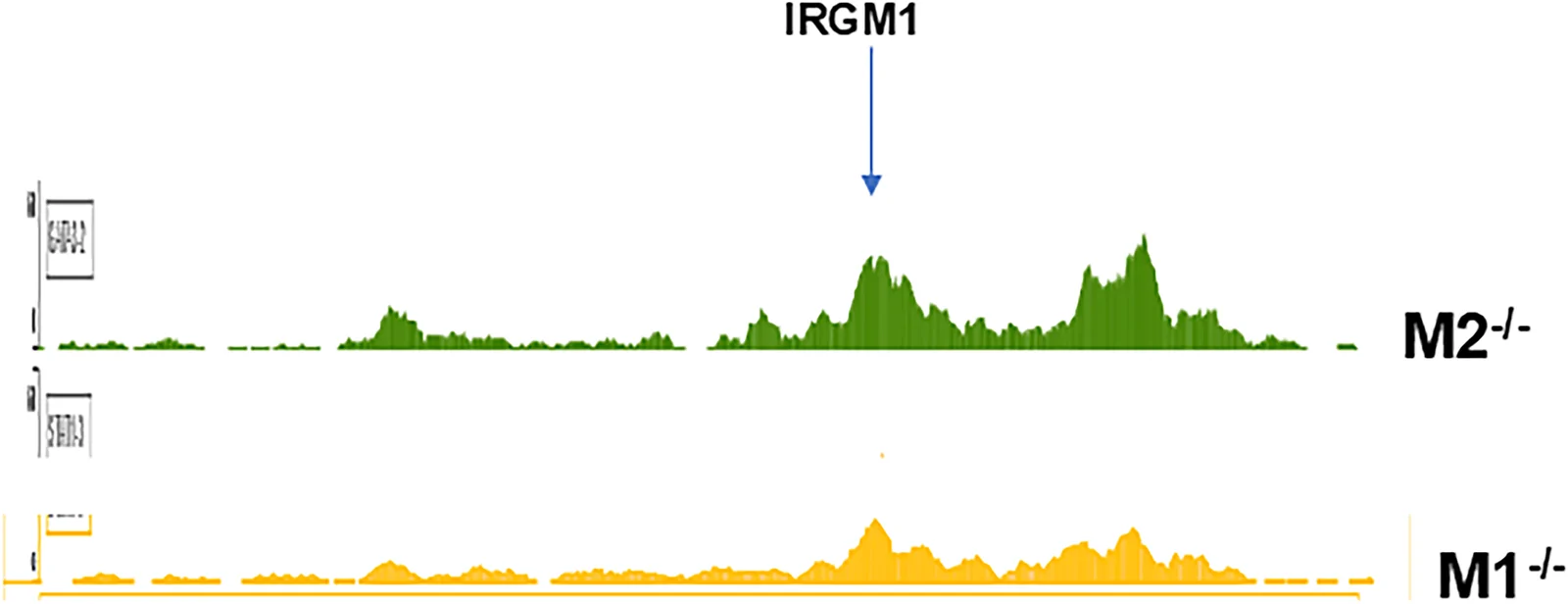
ATAC analysis of IRGM1 in isolated spleen macrophages from latently infected M1^-/-^ and M2^-/-^ mice. M1^-/-^ and M2^-/-^ mice were ocularly infected with 2 X 10^5^ pfu/eye of HSV-1 KOS strain or mock-infected. On day 35 PI, spleens were harvested, dissociated into single-cell suspensions, and red blood cells were removed. Spleens were harvested and dissociated into single-cell suspensions. Cells were stained with 7-AAD, CD3, CD19, Ly6g, F4/80, and CD45 antibodies. CD45^+^F4/80^+^CD3^-^CD19^-^ly6g^-^ macrophages were sorted and subjected to ATAC-seq. IRGM1 peaks in green signify M2^-/-^ macrophages (with M1 macrophages intact), and IRGM1 peaks in yellow signify M1^-/-^ macrophages (with M2 macrophages intact).

To determine if macrophages isolated from the spleens of latently infected M1^-/-^ and M2^-/-^ mice have a similar response as BM-derived macrophages from WT, M1^-/-^, and M2^-/-^ mice, M1^-/-^ and M2^-/-^ mice were infected as above, and spleens were harvested on day 35 PI. Spleen cells were dissociated into single cells, red blood cells were removed, and the cells were plated. The following day, cells were stained with antibodies against 7-AAD, CD3, CD19, Ly6g, F4/80, and CD45. The CD45^+^F4/80^+^CD3^-^CD19^-^Ly-6G^-^ macrophages were sorted and subjected to ATAC-seq. Our data demonstrated that just like the BM-derived M1 and M2 macrophages from WT mice, M1 macrophages from the spleen of M2^-/-^ mice displayed a higher open chromatin peak of IRGM1 as compared to M1^-/-^ mice **(**Fig 3**; compare M2**^**-/-**^ **spleen cells with M1**^**-/-**^ **spleen cells).** These findings confirmed the critical role of IRGM1 in the macrophage memory response and demonstrated that M1 macrophages exhibit a heightened response to HSV-1 training.

### Higher IRGM1 expression in the spleens of latently infected M2^-/-^ mice

**Fig 4:**
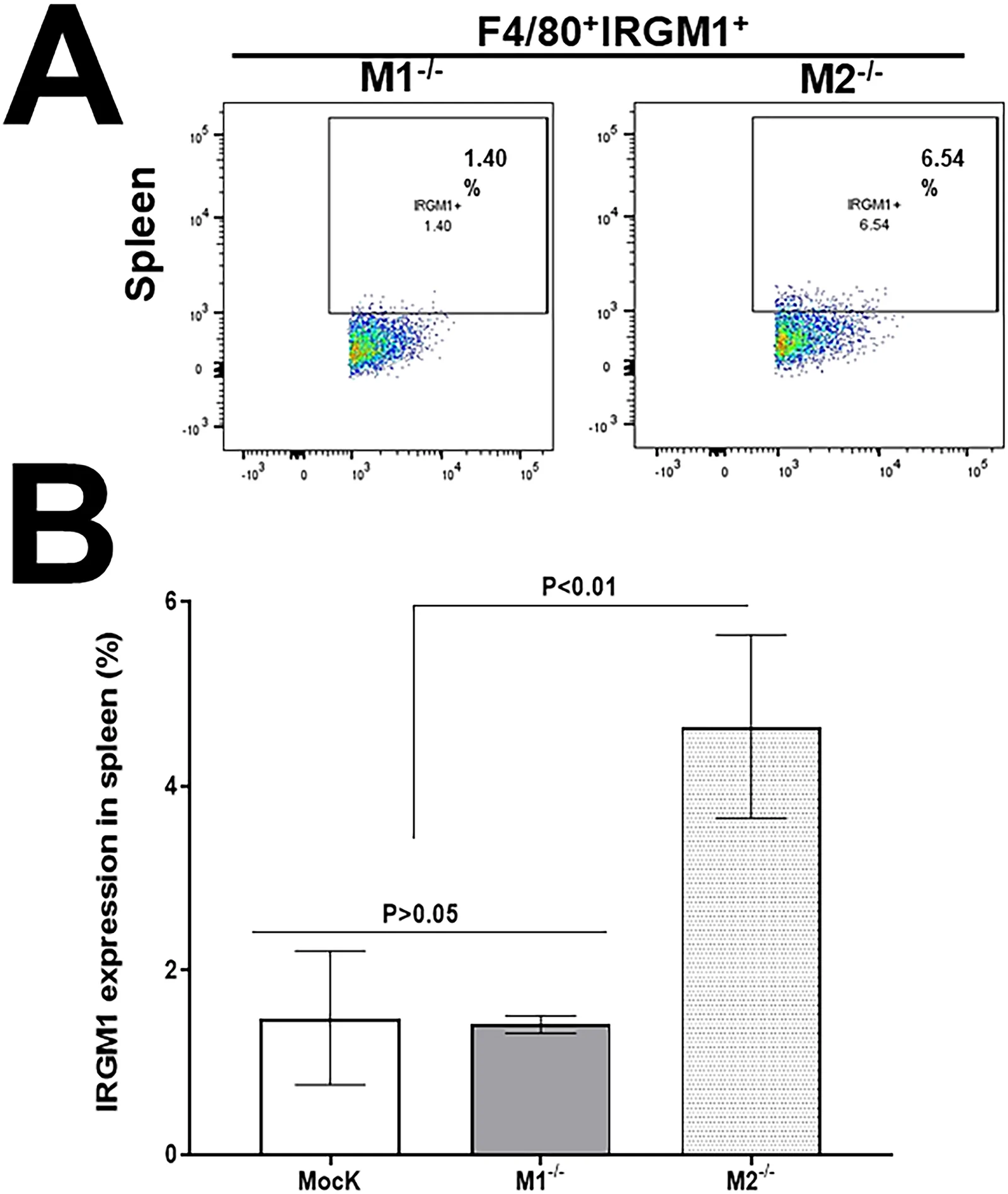
Higher expression of IRGM1 in spleens of latently infected M2^-/-^ mice. M1^-/-^ and M2^-/-^ mice were ocularly infected with 2 X 10^5^ pfu/eye of HSV-1 KOS or mock-infected as described above. On day 35 PI, spleens were harvested and dissociated into single-cell suspensions, and red blood cells were removed. Cells were then plated and stimulated with UV-inactivated HSV-1 (10 pfu/cell) or mock-stimulated for 24 hr. Cells were harvested and stained with the indicated antibodies as described in the Materials and Methods section. Stained cells were gated for CD45^+^F4/80^+^IRGM1^+^Lin^-^, and the percentages of IRGM1^+^ cells were evaluated by flow cytometry (panel **A)**. The average of IRGM1 + cells from 3 separate flow cytometry experiments is shown in panel B in percentage (mean ± SEM).

We next determined differences in IRGM1 expression levels in the spleens of M1^-/-^ and M2^-/-^ mice by Flow cytometry. M1^-/-^ and M2^-/-^ mice were infected ocularly with 2 X 10^5^ of HSV-1 strain KOS with corneal scarification. On day 35 PI, spleens were harvested, dissociated into single cells, and stained with 7-AAD, CD3, CD19, Ly-6G, F4/80, and CD45 antibodies. The CD45^+^F4/80^+^CD3^-^CD19^-^Ly-6G^-^ macrophages were sorted. Sorted macrophages from M2^-/-^ mice (M1 macrophages) showed higher percentages of F4/80^+^IRGM1^+^ cells in comparison to M1^-/-^ mice (M2 macrophages) (Fig 4A**; 6.54% versus 1.40%**). IRGM1 levels in the M1^-/-^ group were comparable to those in mock-treated control (Fig 4B**, P** **> 0.05**). In contrast, the M2-/- group exhibited a marked upregulation of IRGM1 expression, reaching levels more than two-fold higher than those of either the mock or M1-/- mice (Fig 4B**, P** **< 0.01**). These data indicate that M1, but not M2, macrophages participate in the training mechanism in the spleens of latently infected mice.

### Elevated expression levels of IRGM1 in the corneas of latently infected M2^-/-^ mice

**Fig 5:**
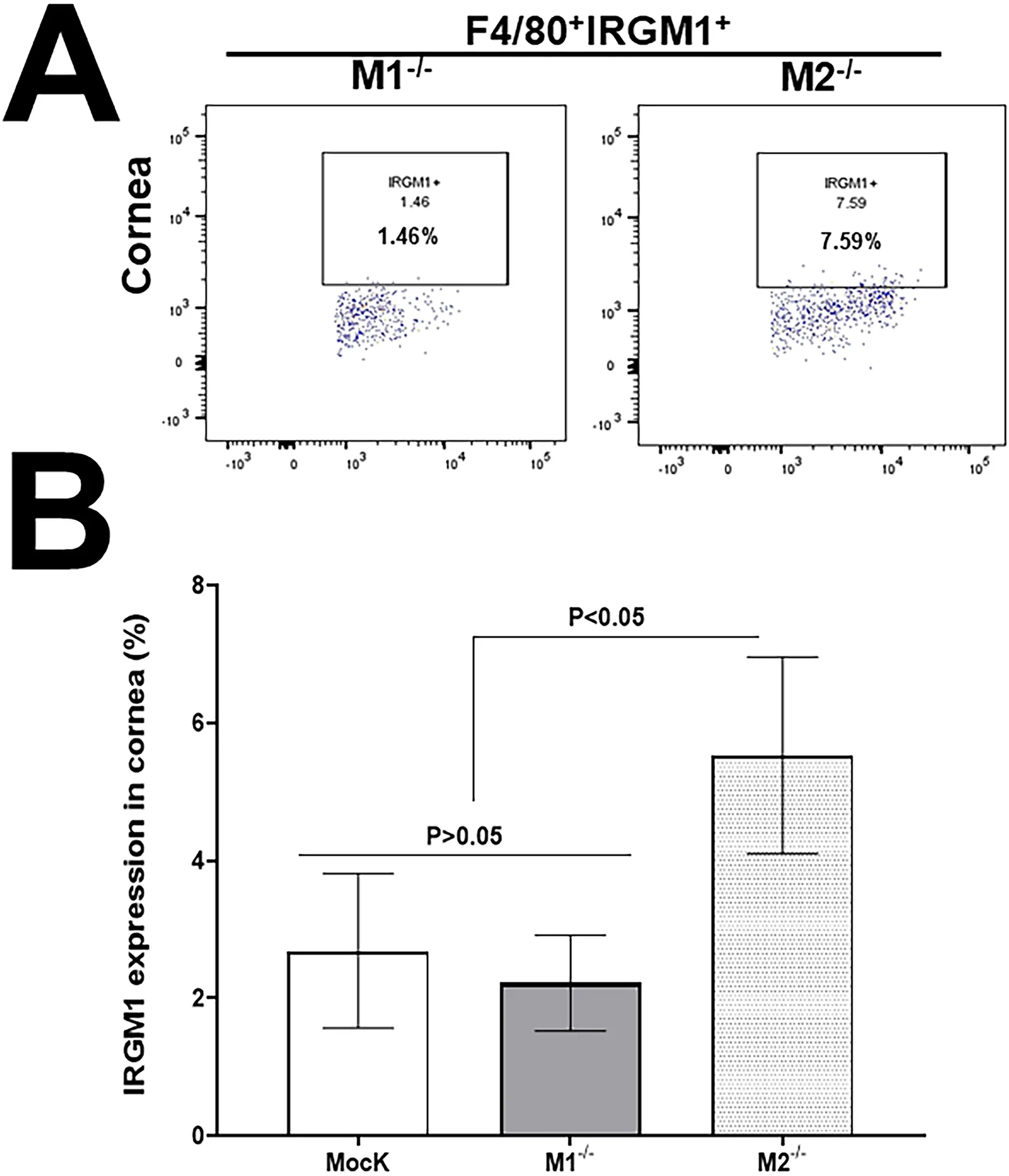
Higher IRGM1 expression in corneas of infected M2^-/-^ mice. M1^-/-^ and M2^-/-^ mice were infected ocularly with 2 X 10^5^ PFU/eye of HSV-1 KOS virus. On day 35 PI, corneas were harvested and dissociated into single cell suspensions. Cells were harvested and stained with the indicated antibodies as described in the Materials and Methods section. Stained cells were gated for CD45^+^F4/80^+^IRGM1^+^Lin^-^, and the percentage of IRGM1^+^ cells was evaluated by flow cytometry (panel **A)**. The average of IRGM1 + cells from 3 separate flow cytometry experiments is shown in panel B as a percentage (mean ± SEM).

Similar to the data from BM-derived and spleen macrophages described above, we next investigated whether IRGM1 expression is affected in the corneas of latently infected M1^-/-^ and M2^-/-^ mice. Mice were infected as above, and corneas were harvested on day 35 PI, dissociated into single cells, and stained for the F4/80^+^IRGM1^+^ cell population. As shown **in** Fig 5A**, IRGM1**^+^ cells in M2^-/-^ mice were significantly increased in comparison to M1^-/-^ mice (**7.59% versus 1.46% cells**). Levels of IRGM1 expression in the M1^-/-^ mice were comparable to those in the mock-treated controls (Fig 5B**, P > 0.05**). In contrast, the M2-/- group showed a significantly higher IRGM1 expression than both the mock and M1-/- groups (Fig 5B**, P < 0.01**). Consistent with our results with spleen macrophages **(**Fig 4**, above)**, these findings suggest that M1, but not M2, macrophages are responsible for macrophage training in response to HSV-1 infection.

### Elevated expression of IRGM1 in the TG of latently infected M2^-/-^ mice

**Fig 6:**
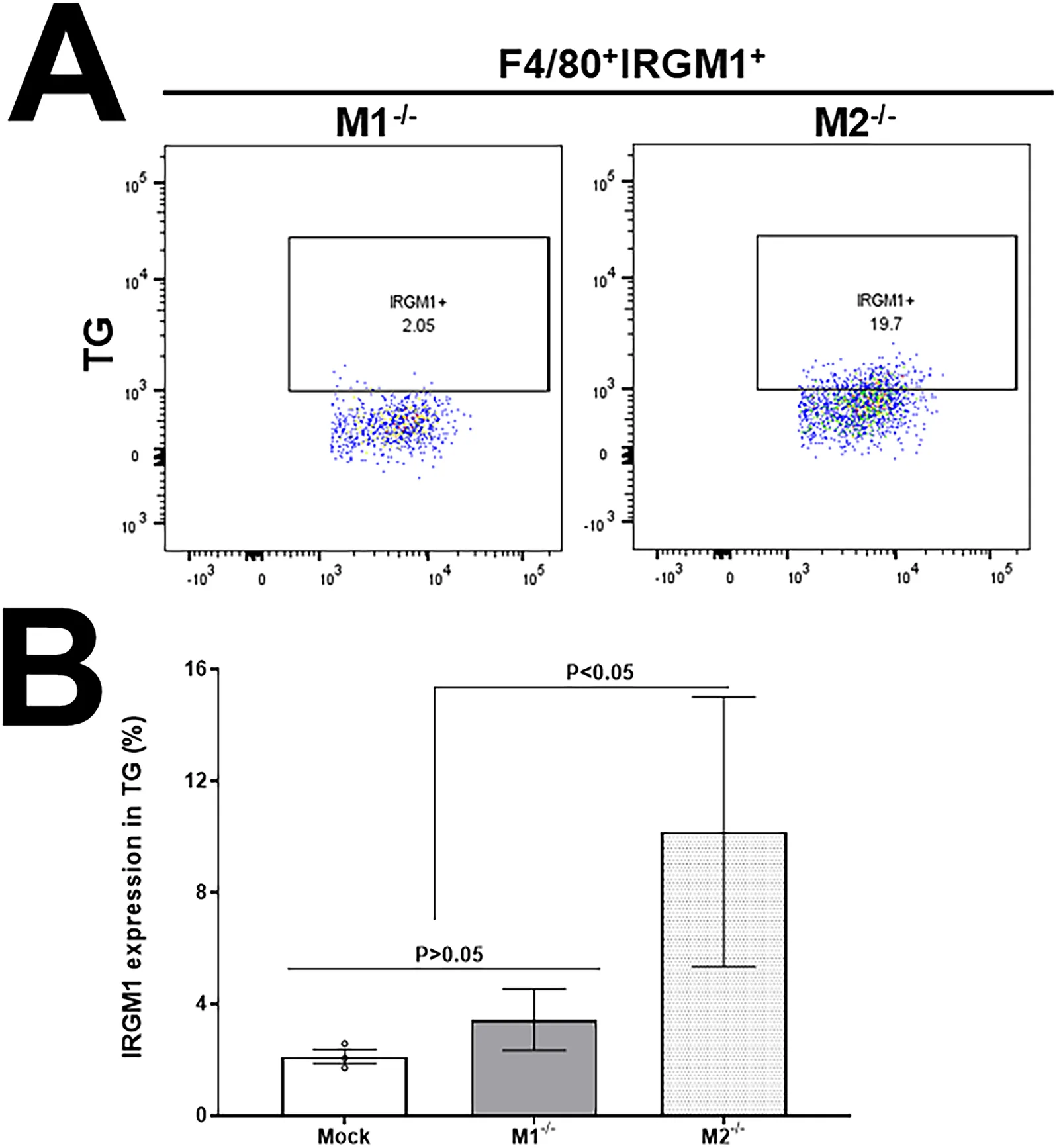
IRGM1 in TG of infected M1^-/-^ and M2^-/-^ mice. M1^-/-^ and M2^-/-^ mice were infected as above. On day 35 PI, TG were harvested, pooled, and dissociated into single-cell suspensions. Cells were then stained with antibodies against CD45, F4/80, IRGM1, and Lin. Stained cells were gated for CD45^+^F4/80^+^IRGM1^+^Lin^-^, and the percentage of IRGM1^+^ cells was evaluated by flow cytometry (panel **A)**. The average of IRGM1 + cells from 3 separate flow cytometry experiments is shown in panel B as a percentage (mean ± SEM).

After initial viral replication and inflammation during primary HSV-1 infection, TG harbors the latent virus [19]. Therefore, we examined whether macrophages in latently infected TG exhibit trained immunity, as evidenced by upregulation of IRGM1 expression. Thus, M1^-/-^ and M2^-/-^ mice were infected ocularly with HSV-1 KOS as described above. On day 35 PI, TG were harvested, dissociated into single-cell suspensions, and subjected to flow cytometry. Cells were stained for F4/80^+^IRGM1^+^ macrophages, and IRGM1 expression was determined in M1 and M2 macrophages. Our data demonstrated that M1 macrophages in M2^-/-^ mice have a higher percentage of IRGM1^+^ expression as compared to M2 macrophages in M1^-/-^ mice (Fig 6A**, 19.7% versus 2.05%**). The levels of IRGM1 expression in the M1^-/-^ mice were comparable to those in mock-treated control (Fig 6B**, P** **> 0.05**), while the M2-/- group showed a significantly higher IRGM1 expression than both the mock and M1-/- groups (Fig 6B**, P < 0.05**). Therefore, similar to our results with spleen, corneas, and BM, M1 but not M2 macrophages in the TG of latently infected mice are trained to HSV-1 infection.

### Chemokine and Cytokine detection in BM-derived M0, M1 and M2 macrophages

Several cytokines and chemokines, including IL-1α, IL-1β, IL-6, IL-10, IL-12, and TNF-α, have been implicated in immune memory responses [20,21]. To investigate the potential contribution of cytokines and chemokines to macrophage training, M0, M1, and M2 macrophages were generated from the BM of WT mice as described in Materials and Methods. Culture supernatants from mock-infected and latently infected macrophages before and after stimulation with UV-inactivated HSV-1 were collected at 24 h post stimulation. Cytokine and chemokine concentrations were quantified using the Illumina Mouse 32-Plex Luminex assay, and the results are summarized in Table 1.

**Table 1:** Selected cytokine/chemokine levels in BM-derived macrophages from latently infected and mock-infected WT mice, measured before and after HSV-1 stimulation^a^.

#	Cytokine/ Chemokine	Mock-infected						Latently infected					
		No stimulation			Stimulated			No stimulation			Stimulated		
		M0	M1	M2	M0	M1	M2	M0	M1	M2	M0	M1	M2
1	G-CSF	3.1 ± 0.58	2.9 ± 1.76	0	55.6 **±** 4.7	31.6 **±** 0.32	26.4 **±** 5.39	1.56 **±** 0.37	0.17 **±** 0.14	1.17 **±** 1.13	175.7 **±** 7.86	51.6 **±** 14.1	19.9 **±** 2.73
2	Eotaxin	3.1 ± 0.58	2.9 ± 1.76	0	55.6 **±** 4.7	31.6 **±** 0.32	26.4 **±** 5.39	10.2 **±** 8.32	1.70 **±** 1.70	2.66 **±** 2.66	0 **±** 0.19	0	4.34 **±** 3.59
3	GM-CSF	10.9 ± 8.49	18.3 ± 9.29	0	41.3 **±** 8.6	36.5 **±** 4.38	35.4 **±** 6.56	5.1 **±** 4.11	58.4 **±** 0.74	4.24 **±** 4.24	50.4 **±** 2.08	58.4 **±** 13.8	26.9 **±** 8.77
4	IFN-γ	1.89 ± 0.75	2.63 ± 1.30	0	6.27 **±** 0.88	5.84 **±** 0.75	4.61 **±** 1.23	0.07 **±** 0.45	58.4 **±** 0.22	1.76 **±** 1.15	9.2 **±** 1.34	9.2 **±** 0.85	5.10 **±** 1.98
5	IL-1α	2.53 ± 0.65	1.70 ± 0.40	0	9.25 **±** 1.01	6.13 **±** 0.38	14.3 **±** 3.61	2.0 **±** 0.37	58.4 **±** 0.68	0.16 **±** 0.00	26.5 **±** 1.69	8.79 **±** 2.77	9.17 **±** 1.63
6	IL-1β	0.33 ± 0.33	0.78 ± 0.78	0	36.07 **±** 2.55	11.4 **±** 0.33	77.1 **±** 5.83	1.46 **±** 0	0	0	204.3 **±** 8.90	23.5 **±** 6.78	59.4 **±** 6.36
7	IL-2	1.50 ± 0.19	1.28 ± 0.22	0.60 ± 0.17	1.96 **±** 0.06	1.34 **±** 0.25	1.76 **±** 0.34	1.60 **±** 0.27	1.05 **±** 0.06	0.98 **±** 0.15	2.42 **±** 0.53	3.33 **±** 0.62	1.44 **±** 0.26
8	IL-4	1.23 ± 0.06	1.10 ± 0.17	0.44 ± 0.04	1.46 **±** 0.10	0.85 **±** 0.05	1.34 **±** 0.38	1.31 **±** 0.06	0.82 **±** 0.14	0.85 **±** 0.18	1.56 **±** 0.15	2.30 **±** 0.66	1.57 **±** 0.26
9	IL-3	1.50 ± 0.24	1.84 ± 0.22	0.35 ± 0.08	2.51 **±** 0.52	1.94 **±** 0.09	2.65 **±** 0.52	1.33 **±** 0.16	0.89 **±** 0.01	0.83 **±** 0.07	2.82 **±** 0.10	3.92 **±** 1.21	2.23 **±** 0.53
10	IL-5	2.09 ± 0	1.74 ± 0.81	0.03 ± 0.03	3.81 **±** 0.81	2.49 **±** 0.54	2.49 **±** 0.54	1.22 **±** 0.54	0.53 **±** 0.26	1.64 **±** 0	3.52 **±** 0.20	4.01 **±** 0.96	2.68 **±** 0.95
11	IL-6	2.10 ± 0.71	3.51 ± 1.92	0	4833.6 **±** 143.6	5387.7 **±** 506.8	5582.3 **±** 460.9	1.95 **±** 1.53	0	0.28 **±** 0	10780.1 **±** 463.21	8117.4 **±** 1365.2	2355.05 **±** 236.5
12	IL-7	2.17 ± 0.53	2.57 ± 0.72	0	2.04 **±** 0.87	1.25 **±** 0.46	1.26 **±** 0.73	1.87 **±** 0	0.92 **±** 0.08	1.08 **±** 0.21	1.18 **±** 0.34	1.94 **±** 0.47	1.39 **±** 0.71
13	IL-9	36.9 ± 23.4	49.0 ± 9.22	10.1 ± 10.1	147.4 **±** 6.90	111.7 ± 5.94	137.9 **±** 23.1	61.3 **±** 9.02	23.5 **±** 7.05	0	189.0 **±** 9.22	159.4 **±** 17.7	126.6 **±** 24.8
14	IL-10	7.52 ± 0.30	2.24 ± 1.12	0	605.9 **±** 23.9	198.0 ± 5.09	245.3 **±** 17.6	2.91 **±** 0	0.26 **±** 0.26	2.60 **±** 0	953.2 **±** 21.05	464.5 **±** 69.6	196.2 **±** 15.2
15	IL-12 (p40)	10.3 ± 2.98	2.30 ± 2.26	0	15.5 **±** 5.57	9.22 ± 1.18	3.08 **±** 3.08	2.89 ± 1.77	4.64 **±** 2.66	3.30 **±** 0	2.86 **±** 0.43	12.6 **±** 6.38	9.94 **±** 5.39
16	IL-12 (p70)	0.37 ± 0.22	1.83 ± 1.07	0	2.82 **±** 1.10	2.33 ± 0.35	3.39 **±** 1.32	1.12 ± 0.14	0.11 **±** 0.11	0.65 **±** 0.49	4.79 **±** 0.85	6.30 **±** 2.18	1.55 **±** 0.78
17	LIF	0.72 ± 0.66	1.27 ± 0.58	0	1.29 **±** 0.98	0.37 ± 0.12	1.37 **±** 0.82	0.39 ± 0.20	0.04 **±** 0.04	0.29 **±** 0	0.69 **±** 0.48	1.54 **±** 1.05	0.50 **±** 0.37
18	IL-13	0	0	0	0	0	0	0	0	0	0	0	0
19	LIX	45.8 ± 5.10	21.06 ± 11.4	0	187.1 ± 7.51	154.5 ± 19.7	125.9 ± 8.05	5.21 ± 6.38	29.4 ± 19.8	50.2 ± 17.8	282.2 **±** 5.57	188.9 **±** 22.7	86.2 ± 5.34
20	IL-15	7.04 ± 5.87	2.63 ± 2.63	0	26.2 ± 4.24	21.3 ± 1.54	26.6 ± 8.90	5.26 ± 1.55	2.54 ± 1.49	3.54 ± 0	31.1 ± 1.77	32.9 ± 5.40	16.4 ± 8.67
21	IL-17	1.91 ± 0.69	2.62 ± 0.67	0	17.8 ± 1.76	11.01 ± 1.47	15.9 ± 1.97	1.25 ± 0.63	0.94 ± 0.22	0.86 ± 0.10	17.4 ± 0.75	17.5 ± 3.64	12.9 ± 1.00
22	IP-10	206.4 ± 24.5	1471.4 ± 4.05	68.4 ± 11.09	15155.14 ± 2740.4	7701.0 ± 434.97	15008.04 ± 3410.5	131.3 ± 2.84	1043.02 ± 122.2	26.2 ± 6.87	20331.8 ± 215.5	16875.91 ± 3971.21	15402.6 ± 3919.2
23	KC	4.82 ± 0.35	5.28 ± 0.55	2.04 ± 0.34	1128.02 ± 104.6	938.12 ± 17.8	673.01 ± 46.1	5.34 ± 0.89	2.47 ± 0.41	2.01 ± 0.37	1519.6 ± 27.1	1090.9 ± 103.7	590.3 ± 15.7
24	MCP-1	1053.4 ± 123.6	18.67 ± 2.93	2022.14 ± 116.7	6941.03 ± 173.7	1712.4 ± 180.0	12391.98 ±944.2	949.7 ± 22.7	13.5 ± 3.28	1249.9 ± 53.1	7983.8 ± 160.5	3728.9 ± 615.4	7332.96 ± 81.5
25	MIP-1α	224.8 ± 26.8	17.9 ± 3.10	106.6 ± 4.97	10121.9 ± 433.05	4146.6 ± 81.5	4858.12 ± 225.3	304.2 ± 1.51	27.94 ± 9.26	116.2 ± 2.27	17581.9 ± 1273.6	17309.2 ± 2444.7	4495.72 ± 317.7
26	MIP-1β	236.3 ± 23.5	60.21 ± 11.72	114.2 ± 6.23	28646.44 ± 1304.59	21968.48 ± 449.5	24879.95 ± 1719.15	295.04 ± 2.17	47.75 ± 9.86	117.4 ± 7.67	22485.4 ± 1472.4	25250.2 ± 1777.2	26859.67 ± 942.9
27	M-CSF	1.83 ± 0.76	3.82 ± 1.47	0	15.21 ± 1.54	11.60 ± 1.66	15.49 ± 2.41	13.8 ± 14.27	2.98 ± 0.73	0.21 ± 0.26	34.8 ± 13.3	16.32 ± 2.89	14.10 ± 2.20
28	MIP-2	0	30.0 ± 0	0	5257.2 ± 266.3	4171.96 ± 340.4	3161.3 ± 132.3	0	4.42 ± 4.42	4.42 ± 0	7270.3 ± 82.23	5227.7 ± 605.83	2272.64 ± 72.54
29	MIG	6.08 ± 0.76	573.15 ± 12.7	0	17729.76 ± 1490.76	3862.73 ± 668.4	5934.88 ± 887.6	6.85 ± 0	513.2 ± 23.38	0.27 ± 0.33	16149.45 ± 1338.8	6191.9 ± 1284.6	1219.5 ± 67.8
30	RANTES	3.87 ± 0.77	22.8 ± 1.26	2.38 ± 0.44	0	0	0	3.25 ± 0.63	18.08 ± 0.75	2.15 ± 0.31	0	0	0
31	VEGF	193.19 ± 17.8	276.51 ± 29.4	236.85 ± 21.89	26.2 ± 3.58	67.21 ± 8.19	8.78 ± 0.77	205.8 ± 19.9	115.03 ± 5.62	175.5 ± 5.27	40.27 ± 3.84	57.17 ± 9.30	14.96 ± 1.75
32	TNF-α	3.29 ± 0.34	2.00 ± 0.12	1.62 ± 0.12	797.20 ± 93.78	1533.6 ± 126.4	187.7 ± 27.61	4.07 ± 0.64	1.81 ± 0.22	2.14 ± 0.46	1655.8 ± 89.5	16071.3 ± 6953.9	241.19 ± 25.5

a Cytokines and chemokines levels in the culture media were analyzed using mouse 32-plex panels, and the level of cytokine or chemokine is shown as pg/ml. BM-derived macrophages from mock-infected and latently infected WT C57BL/6 mice were used in the experiment. Details of the experimental procedures are described in Materials and Methods section. Briefly, mice were infected ocularly with 2 X 10^5^ pfu/eye of HSV-1 strain McKrae without corneal scarification. Isolated BM cells were exposed to either IFN-γ with LPS or IL-4 and allowed to undergo M1 and M2 activation, respectively, and the M0 group was mock-treated. After 24 hr of exposure, some of the cells were treated with 10 pfu/cell of UV-inactivated HSV-1 strain McKrae for 1 hr at 37°C, washed with PBS, and incubated for an additional 24 hr in fresh media. Results indicate mean ± SEM.

As shown in Table 1, the concentrations of G-CSF, IL-1α, IL-1β, IL-6, IL-9, IL-10, LIX, KC, MCP-1, MIP-1α, MIP-1β, MIP-2, MIG, and TNF-α were consistently higher in stimulated macrophages than in unstimulated macrophages in both mock-infected and latently infected groups. Among all conditions, these cytokines and chemokines reached their highest levels in stimulated M1 macrophages derived from latently infected mice. These findings are consistent with the established pro-inflammatory phenotype of M1 macrophages and suggest that latent infection further enhances their inflammatory response following stimulation. In contrast, Eotaxin expression was markedly increased in stimulated macrophages from mock-infected mice but remained low in macrophages from latently infected mice, regardless of stimulation. IFN-γ expression was highest in unstimulated M1 macrophages derived from latently infected mice (Table 1).

IL-2, IL-3, IL-4, IL-5, IL-7, IL-12 (p70), and LIF were detected at low levels across all experimental groups Table 1). IL-13 remained below the limit of detection in all samples and was not affected by either stimulation or latent infection. M-CSF levels were highest in stimulated M0 macrophages from latently infected mice. Because M-CSF was included in the culture medium for both stimulated and unstimulated macrophages, the increased levels observed following stimulation may reflect enhanced production or accumulation by M0 macrophages from latently infected mice rather than differences solely attributable to the exogenously added cytokine. RANTES was below detectable levels in stimulated M0, M1, and M2 macrophages from both mock-infected and latently infected mice but was detectable in the corresponding unstimulated macrophage populations. Conversely, VEGF expression was consistently higher in unstimulated macrophages than in stimulated macrophages regardless of infection status.

Overall, these data demonstrate that stimulation induces robust inflammatory cytokine and chemokine responses, particularly in M1 macrophages derived from latently infected mice, whereas several growth factors and regulatory mediators, including VEGF and RANTES, exhibit an opposing pattern of expression. These findings suggest that latent infection selectively amplifies pro-inflammatory macrophage responses while altering the production of factors involved in tissue repair and immune regulation.

## Discussion

Macrophages are essential cells of the innate immune system that show functional diversity [22]. We have previously shown that, following ocular infection in immunized mice, macrophages are the most abundant infiltrating cells in the mouse cornea [23,24]. We have also shown that macrophage depletion increases viral replication in the eyes and TG of immunized mice following primary ocular HSV-1 infection [13]. Therefore, they not only provide the initial defense against microorganisms but also initiate and control adaptive immune responses. Cellular infiltrates that are detectable in the corneas of mice ocularly infected with HSV-1 have been implicated in the disease process [24–30]. These infiltrates appear to play distinct roles in the disease process depending on the environmental milieu, including the abundance and characteristics of the infiltrates, the infection dose, and the mouse model (strain of mouse, strain of virus, and whether infection is performed with or without corneal scarification). Thus, complex interactions between HSV-1 and infiltrating immune cells play essential roles in establishing both localized acute viral replication and chronic latent infection. Although it is evident that macrophages likely play a central role in acute and chronic HSV-1 infection, it is also apparent that they can act to either exacerbate or protect against these processes [13,31–34]. Macrophages are mononuclear phagocytes that are important for tissue homeostasis and the resolution of inflammation and have been identified as crucial players in the elicitation of potent antiviral immune responses [35,36]. Innate immune memory macrophages have also been reported to confer resistance against S. aureus skin infections [37].

Macrophages can alter their phenotype in response to environmental signals [38]. Polarization is often viewed on a simplified, linear scale, in which M1 (classically polarized) macrophages represent one extreme and M2 (alternatively polarized) macrophages the other (36), with the phenotype of polarized macrophages reflecting a delicate equilibrium between the M1 and M2 profiles [39]. M1 macrophages, after induction by proinflammatory mediators, produce significant amounts of proinflammatory cytokines (TNF-α, IFN-γ, IL-6, and IL-12) and generate reactive oxygen species, including nitric oxide through activation of inducible nitric oxide synthase (iNOS) [11,12]. In contrast, M2 macrophages, which are induced by exposure to IL-4, IL-13, IL-10, or glucocorticoids, do not secrete high levels of proinflammatory cytokines but produce high levels of anti-inflammatory cytokines (IL-10, TGF-β, and IL-1 receptor antagonist), as well as the enzyme, arginase 1 (ARG1) [12,40]. Thus, M2 macrophages are believed to participate in the blockade of inflammatory responses and to promote tissue repair/angiogenesis as well as T_H_2 immune responses [11].

In our recent publication, we demonstrated that macrophages are trained against HSV-1 infection [6]. Our data showed upregulation of IRGM1 expression in infected corneas, spleen macrophages, TG, and BM-derived macrophages compared with naive tissues. IRGM1 is a member of the Immunity-related GTPases (IRGs) and regulates membrane remodeling events in cells, including autophagy and mitophagy [41]. IRGM1 is the only gene involved in protection against a broad range of pathogens, unlike other IRG members, which are known for destroying mycoplasma [17]. Additionally, IRGM1 deficiency has been linked to an increased risk of various autoimmune diseases [42]. In a study conducted with uninfected IRGM1-deficient mice and cultured IRGM1-deficient macrophages, the authors reported that mice exhibited elevated serum cytokine levels, leading to enhanced autoinflammation, and that this was also observed in cultured macrophages [43]. In another study, the authors reported that type 1 IFN signaling and metabolic dysfunctions, such as enhanced glucose metabolism in macrophages, contribute to robust inflammatory cascades in IRGM1-deficient macrophages [43,44]. The human IRGM gene has been strongly associated with Crohn’s disease and other inflammatory diseases through Genome-Wide Association studies [41].

Therefore, building on our recent study [6], we sought to determine which macrophage subpopulation is responsible for the training/memory response. Using a combination of *in vitro* M0, M1, and M2 macrophages from WT mice and M1-M2 Knockout mice models, we demonstrated that M1 macrophages play a prominent role in training macrophages to respond to HSV-1 infection. Our data, as determined by ATAC-seq, displayed a much higher peak in infected BM-derived macrophages, corneas, spleen macrophages, and TG in M2^-/-^ mice in comparison to M1^-/-^ mice. One interesting finding, and consistent with other studies, is that only stimulated M1 macrophages displayed enhanced memory characteristics after HSV-1 infection. This may be because, in unstimulated macrophages, chromatin regions containing inflammatory genes are compacted, making transcriptional access difficult. Upon re-challenge with the same antigen or a different one, chromatin shows increased DNA methylation and histone modifications that facilitate transcription [45]. Thus, as reported in other studies, epigenetic and metabolic changes occur during immune cell stimulation [46,47]. Along with that, our Luminex data demonstrate that the increased production of inflammatory mediators, including IL-1α, IL-1β, IL-6, TNF-α, G-CSF, MCP-1, MIP-1α, MIP-1β, MIP-2, KC, LIX, and MIG, is consistent with a trained or memory-like macrophage phenotype in which prior infection results in heightened responsiveness to subsequent stimulation. This increase in cytokine and chemokine expression in M1 macrophages, as well as increased IRGM1 expression in M1 macrophages, strongly suggests a link between trained immunity and a recall response. These molecules are functional outputs, not defining markers, of memory macrophages. The cytokines/chemokines listed above reflect the M1 macrophage’s ability to orchestrate the recruitment of other immune cells during the recall response. Previous studies have demonstrated that secondary stimulation of trained macrophages results in enhanced production of IL-1β, TNF-α, IL-6, and inflammatory chemokines, reflecting epigenetic and metabolic reprogramming that promotes heightened innate immune responsiveness [20,48]. Therefore, the cytokine and chemokine profile observed in stimulated M1 macrophages from latently infected mice is consistent with a memory-like (trained) macrophage phenotype characterized by an amplified pro-inflammatory response upon restimulation. On further investigation of the underlying epigenetic mechanisms and pathways regulated by M1 and M2 stimulated macrophages using ATAC-seq, interestingly, in stimulated M1 macrophages, pathways associated with T_H_1 and T_H_2 activation, T_H_1 signaling, and key pro-inflammatory mediators, including STAT1, IFN-γ, TNF-α, and IL-1β were downregulated (not shown). This apparent contradiction is a common observation when comparing Luminex and ATAC-seq, because the two assays measure very different aspects of the inflammatory response, where Luminex measures secreted proteins (cytokines) in the culture supernatant, and ATAC-seq measures chromatin accessibility, which reflects the potential for gene transcription, not protein production.

This suggests a shift toward an anti-inflammatory or regulatory phenotype despite the enhanced memory response. Recently, it was shown that IFN-γ plays a role in memory formation in human macrophages [49]. In our study, IFN-γ was not involved in the development of a training response to HSV-1 infection, despite being upstream of the IRGM1 promoter region.

Additionally, we identified differential regulation of immune-associated genes, including Bhlhe40, CD40L, IFN-γ, IL-2, and STAT1, which are known to influence T_H_1/T_H_2 differentiation via the T_H_1/T_H_2 activation pathway, thereby shaping the overall immune response [50–52]. On the other hand, we found that IRGM1 expression in M2-stimulated macrophages was downregulated. This perhaps explains why M2 macrophages play no role in training macrophages to respond to HSV-1 infection. In another study, we demonstrated that M1 macrophages are also responsible for CNS demyelination, whereas M2 macrophages play a protective role, as evidenced by the absence of demyelination plaques [6,53]. Furthermore, we have shown that due to stimulation of macrophages, immunization of mice with a recombinant HSV-1 expressing murine IL-12p35 was more effective in preventing eye disease and reducing virus replication and latency in the eye than immunization with HSV-1 recombinant viruses expressing IL-2, IL-4, IFN-γ, or IL-12p40 [54–57]. However, macrophage depletion caused demyelination in the brain, spinal cord, and optic nerves of mice ocularly infected with different HSV-1 strains [58].

Macrophages are known to regulate innate immunity against many bacterial and viral infections, and memory macrophages in these murine models displayed increased monocyte recruitment, enhanced bacterial killing, and conferred resistance to secondary infections [59,60]. These adaptations are thought to be mediated, at least in part, by epigenetic reprogramming and metabolic rewiring, which enable a more rapid and robust response upon re-exposure to pathogens. In addition to pathogen clearance, trained innate immune cells play a pivotal role in regulating key cellular processes, such as maintaining tissue homeostasis and fine-tuning inflammatory responses. For instance, macrophage training has been associated with sustained changes in cytokine production, including increased secretion of pro-inflammatory mediators and enhanced responsiveness to secondary stimuli, while also preserving mechanisms that prevent excessive tissue damage [61,62]. This balance between heightened responsiveness and controlled inflammation is critical for effective host defense.

Within this context, M1-polarized macrophages are of particular interest due to their potent antiviral and pro-inflammatory properties. Our findings provide fundamental insights into the contributions of M1 macrophages to trained immunity in HSV-1 infection. Specifically, M1 macrophages appear to establish a primed immunological state that enhances antiviral defense mechanisms upon subsequent viral challenge. Taken together, these results highlight the importance of macrophage-mediated trained immunity as a key component of host defense against HSV-1. Further investigation into the molecular pathways underlying this phenomenon could uncover novel therapeutic targets and inform the development of innovative strategies to prevent or treat herpes simplex keratitis (HSK), a leading cause of infectious blindness worldwide.
